# S01 Providing policymakers with the evidence and practices to act and improve lives through physical activity

**DOI:** 10.1093/eurpub/ckac093.001

**Published:** 2022-08-29

**Authors:** Elena Portas, Charlie Cowen, Lottie Birdsall-Strong, Chris Scott

**Affiliations:** 1 Portas Consulting, London, United Kingdom; 2 Data & Insight, Portas Consulting, London, United Kingdom; 3 The Football Association, England, London, United Kingdom; 4 Communications, London Sport, London, United Kingdom

**Keywords:** Social Value, Data and Insight, Decision-making, Collaboration

## Abstract

Policymakers often lack the evidence to drive change and improve the lives of citizens through physical activity. This symposium will discuss how two unique global programs, ‘Active Citizens Worldwide' (ACW) and ‘Sport Impacts: Children' (SIC), have bridged the gap between academia and policymakers. Combining advanced data analytics, the latest in health, social and economic research and global benchmarking these programs have delivered unique insights on the drivers and outcomes of physical activity.

ACW is a city-based programme formed through a collaboration of 4 cities: London, Stockholm, Singapore and Auckland. The project uses advanced analytics on large datasets covering entire cities to inform policymakers' efforts to enhance participation levels, and to calculate the monetary and non-monetary value that sport and physical activity has on society. Previously unrelated datasets have been integrated to provide completely new analytical insights on the true drivers of the complex physical activity system including facility access, and mindsets and motivations.This is combined with systematic modelling of the broad range of outputs and outcomes of physical activity including health, social, and economic benefits.This standardisation in modelling approach and analysis has enabled multiple cities to compare, benchmark and learn from each other. ACW ultimately enables cities to understand what is working and what is not in their city, and thereby design policies with the best chance of improving their city's health, wealth and wellbeing.

Using a similar approach, the SIC initiative is a unique cross-sectorial collaboration between top national sports bodies, charities and academics in England. Through this partnership each organisation shared national participation, workforce and facility databases as well as recent primary research, which was analysed and modelled to give a holistic overview of the drivers and outcomes of childhood participation in sport. This analysis shows that the access to and benefits of sport and physical activity is not equal between different demographic groups. Preliminary findings have also indicated which type of sports and activities contribute the most value to children and their communities. This has led to the creation of a strong evidence base of the social and economic impact of childhood physical activity and sport in England, which is already being used by sport bodies and policymakers to re-evaluate their approaches to childhood participation.

These programs illustrate how a collaborative approach using data and analytics has connected research with policy and practice and lead to substantial impacts in countries around the world.

Organizer and speaker: Elena Portas, Chair: Charlie Cowen,

